# From Whence Cometh My Help? Psychological Distress and Help-Seeking in the Evangelical Christian Church

**DOI:** 10.3389/fpsyg.2021.744432

**Published:** 2021-12-16

**Authors:** Christopher E. M. Lloyd, Graham Reid, Yasuhiro Kotera

**Affiliations:** ^1^Human Sciences Research Centre, University of Derby, Derby, United Kingdom; ^2^Department of Experimental Psychology, University of Oxford, Oxford, United Kingdom

**Keywords:** help-seeking, psychological distress, religion, Evangelical, sin, fundamentalism

## Abstract

Seeking professional help for psychological distress is generally associated with improved outcomes and lower levels of distress. Given the saliency of religious teachings, it has been shown that aspects of Christian belief may influence adherents’ attitudes towards mental health help-seeking. Based on existing research on American Evangelicals, it was hypothesised that religious social support would positively predict attitudes towards mental health help-seeking, whilst fundamentalism, mental distress, and the belief that psychopathology is caused by immoral or sinful living would negatively predict participants’ attitudes. On a convenience sample of 252 British Evangelicals, our hypotheses were supported and these variables significantly predicted participants’ attitudes towards seeking mental health help, *F*(7,243) = 9.64, *p* < 0.001, *R*^2^ = 0.195. These findings together suggest that whilst religious support positively predicts help-seeking attitudes, Evangelical fundamentalism, in addition to beliefs that mental illness has a spiritual cause, as well as experiences of mental distress may be associated with more negative attitudes towards psychotherapeutic intervention. Thus, mental health practitioners should be aware of clients’ religious worldviews and tailor interventions appropriately, acknowledging that working with religious organisations may yield the most positive outcomes for patients.

## Introduction

With evidence of increasing rates of mental illness (Calling et al., 2017; Patalay and Gage, 2019), there is growing concern over the personal and socioeconomic impact of psychological distress (McDaid et al., 2019; Ausín et al., 2020). A key factor in alleviating the undesirable outcomes associated with mental illness is early therapeutic intervention (Cook et al., 2017). Research has shown that supportive attitudes towards mental illness and help-seeking are indispensable in mitigating barriers against service utilisation (ten Have et al., 2010; Mojtabai et al., 2016). However, it seems that a sizable proportion of the adult population have limited ontological and aetiological knowledge of psychiatric problems, which negatively influences their help-seeking attitudes (Jorm, 2000; Rüsch et al., 2011). As a result, many who experience poor mental health do not access treatment (Araya et al., 2018) despite the finding that psychotherapeutic care is efficacious for a number of mental health conditions (McAleavey et al., 2019). Taken together, understanding the factors that influence people’s attitudes towards therapeutic interventions is important in tackling barriers against service non-utilisation.

Although other psychological variables play a role, culture and social identity strongly shape and influence our attitudes (Hogg and Smith, 2007). A particularly potent form of social identity is religion in which adherents have regular interaction with like-minded persons who affirm the validity of their in-group (Mavor and Ysseldyk, 2020). Such access to rich social resources has been suggested as one of the ways in which religious engagement has a buffering effect on the emergence of psychological distress (Hovey et al., 2014; Holt et al., 2018). Not only do members of the same religion benefit from social engagement in times of crisis, but feeling supported by one’s religious community has also been associated with more positive attitudes towards using mental health services (Miville and Constantine, 2006). As a consequence, religion seems to provide a powerful social identity whose behavioural norms and beliefs can influence the extent to which individuals are likely to come forward for help when experiencing distress. Lloyd (2021), for example, claims that religious belief systems frequently carry ontological essences or “seedling psychologies” in that they offer specific frameworks for making sense of illness, disease, and life more generally, illustrating the strong impact of religion on one’s attitudes.

However, religion is not a unified construct that can be consistently associated with more positive attitudes towards psychological interventions (Shadid et al., 2021). For example, a particular instantiation of Christianity known as Evangelicalism may promote reductive theological axioms, which discourage favourable attitudes towards psychotherapeutic participation (Lloyd and Waller, 2020). Although the definition is variable, Evangelicalism is a Protestant *trans-*denominational tradition, characterised by four main doctrines with over 600 million global adherents (Bebbington, 2003; Pew Research Centre, 2015). The main principles of Evangelicalism are the inerrancy of the Bible, a literal interpretation of scriptures, an exclusive soteriology through faith in Jesus Christ, and the importance of converting non-believers to this theological perspective through a personalised process of regeneration (i.e., being born again). In light of their fundamentalist theology, Evangelicals often conceptualise mental health as vertically representative of their spiritual life, which is not contingent on biopsychosocial mechanisms (Hartog and Gow, 2005).

Such a reductive understanding of mental illness has pitted secular and spiritual care against each other for many believers (Wesselmann et al., 2015). Research has suggested that Evangelicals are reluctant to seek professional help for psychological trouble and instead prefer support from their religious leaders (Chalfant et al., 1990). Indeed, the very act of soliciting professional help rather than trusting in God’s provision may be interpreted as a sign of spiritual weakness or failure (Mayers et al., 2007). Within the context of such an ontology, individuals with psychological distress may have their lived experiences invalidated or ignored (Lloyd, 2021). Lloyd (2021, p. 2719) referred to this negative aspect of religion as “reductive spiritualisation”: a process through which mental health problems may be connected solely with spiritual aspects (demons, sin, or generational curses), with relative neglect towards life context and experience. Furthermore, a recent qualitative study by Lloyd and Hutchinson (2021) also found that Evangelicals with lived experience of psychological ill health reported being socially ostracised and relationally disconnected from their fellow believers. As such, the Evangelical fundamentalist worldview in which mental illness is seen as the result of sinful living may be particularly detrimental as it encourages believers to seek a spiritual solution and be dismissive of secular interventions. Within the context of Evangelicalism, immoral or sinful living might be characterised by a lack of faith, diminished church attendance, lack of repentance of sins, and reduced prayer or devotional life. Scrutton (2020) argues that exclusively spiritual accounts may often be harmful as they potentially de-politicise the social, relational, and political context of mental distress and over direct attention to individual responsibility.

That being said, the majority of attitudinal research towards mental health help-seeking has been conducted on Evangelicals in America. Given these findings do not necessarily transfer to religious groups outside of the United States, this study aimed to investigate the relative contributions of (i) belief that mental illness is caused by immoral or sinful living, (ii) fundamentalism, (iii) religious support, and (iv) psychological distress in predicting attitudes towards psychotherapeutic participation (outcome variable) in United Kingdom Evangelicals. It was hypothesised that each explanatory variable would negatively predict attitudes towards help-seeking in United Kingdom Evangelicals, except religious support, which would be positively related to the outcome variable.

## Materials and Methods

### Ethics

The procedures of the current study were approved by the Ethics Committee at the University of Derby (ETH2021-0070; see Lloyd and Kotera, 2021). Prior to engaging with the study, all participants provided informed consent. Given the online nature of the study, participants could withdraw their consent at any time by closing their browser and up to one week following study completion. Data were anonymised and stored on a GDPR-compliant server to which the researchers had exclusive access. In light of the potentially distressing research topic, information pertaining to mental health charities and helplines were provided in the debrief. The study adhered to the Strengthening the Reporting of Observational Studies in Epidemiology (STROBE) reporting guidelines throughout (von Elm et al., 2007).

### Participants

Participants self-identified as Evangelical Christians and were recruited from online faith communities across the United Kingdom. An *a priori* power analysis estimated a minimum sample size of 119 participants based on α = 0.05, power = 0.95, and *f*^2^ = 0.15 (G*Power 3.1; Faul et al., 2009). After recruiting a sample of 265 participants, a total of 252 (Male = 185; Female = 62; Non-Binary = 5) aged between 18 and 73 (*M* = 46.11 years, *SD* = 13.72) were included in our analyses. Thirteen were not included in the final sample due to missing data or not providing informed consent; however, no reason for withdrawal was asked per our ethical guidelines and no complaint was received by any participants. Regarding the representativeness of our sample, scarcely available data from the Office for National Statistics suggest that 43% of United Kingdom Christians are over 50 years old with a further 31% being between the ages of 25 and 49 (Serafino, 2020). As for gender, data from the Pew Research Centre suggest that women in the United Kingdom are more likely to rate religion as *very important* (25 vs. 18%), to pray daily (23 vs. 14%), and to attend weekly services (15 vs. 10%) compared to men (Hackett et al., 2016). Compared with the general population, the mean age was similar, but the ratio of males was larger in our sample. Participants were not recompensed for study participation.

### Measures

#### Help-Seeking Attitudes

The Inventory of Attitudes Towards Seeking Mental Health Services was used to assess participants’ help-seeking attitudes (IASMHS; Mackenzie et al., 2004). IASMHS consists of 24 items measured on a five-point Likert scale ranging from *disagree* (0) to *agree* (4). The questionnaire has a tridimensional structure, including *psychological openness, help-seeking propensity*, and *indifference to stigma.* In the current study we used participants’ overall scores because we did not have any theoretical justification to focus on a specific dimension. Example items included “if I were experiencing a serious psychological problem at this point in my life, I would be confident that I could find relief in psychotherapy” and “I would feel uneasy going to a professional because of what some people would think.” Participants could score between 0 and 96 on the IASMHS in which higher scores indicated more positive attitudes towards mental health help-seeking. IASMHS has been validated for construct and concurrent validity in a recent study by Hyland et al. (2015). In the current study, IASMHS’s items were internally consistent (α = 0.82) above the field’s minimally accepted standard of 0.70 (Taber, 2018).

#### Religiosity

To assess individual differences in participants’ religiosity, we used the 10-item Religious Commitment Inventory (Worthington et al., 2003). Participants responded to the items on a five-point Likert scale, ranging from *not at all true of me* (1) to *totally true of me* (5). Example items included “my religious beliefs lie behind my whole approach to life” and “I often read books and magazines about my faith.” Total scores ranged from 10 to 50 in which higher scores represented greater levels of religiosity. In the current sample, the items were internally consistent and closely related in their measurement of religious commitment (α = 0.88).

#### Psychological Distress

To measure participants’ experiences of psychological distress over the past 2 weeks, the four-item Patient Health Questionnaire for Anxiety and Depression was used (Kroenke et al., 2009). Participants indicated how often they had experienced psychological distress on a four-point Likert scale, ranging from *not at all* (0) to *nearly every day* (3). Total scores ranged from 0 to 12 in which higher scores were indicative of more frequent experiences of psychological distress over the past 2 weeks. In the current sample, the items were closely related in their operationalisation of psychological distress (α = 0.88).

#### Religious Support

The multidimensional Religious Support Scale was used to assess the extent to which participants experienced a sense of support from their congregation, religious leaders, and God (Fiala et al., 2002). Each subscale has seven-items, leading to a 21-item questionnaire that is measured on a five-point Likert scale. Responses ranged from *strongly disagree* (1) to *strongly agree* (5), resulting in a total score of 21–105 where higher scores indicated a greater sense of religious support. Example items included “my church leaders care about my life and situation” and “I feel appreciated by God.” Previous research has shown that this questionnaire has good criterion validity in predicting mental health outcomes (Willoughby et al., 2008). In the current sample, excellent reliability was observed (α = 0.94).

#### Fundamentalism

The Christian Fundamentalist Belief Scale was used to assess the extent to which participants affirmed the inerrancy and authority of the Bible (i.e., the key facet of Protestant fundamentalism; Gibson and Francis, 1996a,b). Participants responded to the 12-items on a five-point Likert scale, ranging from *strongly disagree* (1) to *strongly agree* (5). Example items included “I believe that the Bible is the word of God” and “I believe in hell.” Total scores on the questionnaire ranged from 12 to 60 in which higher scores represented more fundamentalist beliefs in our sample of Evangelical Christians. In the present study, high reliability was observed (α = 0.88), which demonstrated that the questionnaire’s items consistently measured the underlying fundamentalism construct.

#### Psychiatric Pathogenesis

To assess participants’ aetiological beliefs, we used the Morality-Sin subscale of the Religious Beliefs about Mental Illness Questionnaire (Wesselmann and Graziano, 2010). This nine-item subscale operationalises the extent to which respondents believe that mental illness can be attributed to immoral or sinful living. Example items included “moral weakness is the main cause of mental illness” and “mental illnesses are a result of original sin.” Participants indicated their responses on a nine-point Likert scale, which ranged from *strongly disagree* (1) to *strongly agree* (9). Scores could range from 9 to 89 in which higher scores were indicative of a greater propensity towards attributing the cause of mental illness to immoral lifestyles. The questionnaire demonstrated high reliability in the current sample (α = 0.85).

### Procedure

The present study was disseminated using Microsoft Forms, which was licenced by the University of Derby. Participants were asked to provide informed consent and some demographic information (i.e., age, gender, religious affiliation, and the frequency of their engagement in religious activities). Afterwards, participants completed the psychometric measures of our key variables in a randomised order. To determine participants’ familiarity with mental illnesses, participants were then asked to indicate the capacity in which they gained this knowledge (e.g., media, courses, or lived experience). To assess their commitment to Evangelical Christianity, participants were asked to endorse creedal statements that aligned with Stanford and McAlister (2008)’s research definition of Evangelicalism. Untimed breaks were given throughout the study, which had a mean completion time of 27 min.

### Data Analysis

Data wrangling and statistical analyses were conducted using the statistical language R (Version 3.6.3). All collected data were screened for outliers and the assumptions of our parametric tests were assessed and satisfied. In the current study, we ran a multiple linear regression with a cross-sectional design. Our criterion variable was help-seeking attitudes as measured by scores on the Inventory of Attitudes Towards Seeking Mental Health Services (Mackenzie et al., 2004). We controlled for age, gender, and religious commitment as covariates before assessing the relevant contribution of our predictor variables in explaining the variance in the criterion. The predictor variables in our multiple regression were religious support, fundamentalism, psychological distress, and belief that mental illness is caused by immoral or sinful living.

## Results

Results revealed that after controlling for the demographic covariates, our four predictor variables explained 18.3% of the variance in participants’ attitudes towards seeking mental health help, *F*(7,243) = 9.64, *p* < 0.001. The main contributors of the variability in our criterion in descending order were psychiatric pathogenesis beliefs that mental illness is caused by immoral or sinful living, fundamentalism, psychological distress, and religious support (for a summary of descriptives, see Table 1). Among them, religious support was positively correlated with attitudes towards help-seeking scores. For a full exposition of the model’s parameters, see Table 2.

**TABLE 1:** Descriptive summary of the questionnaire variables.

	*M*	*SD*	α
Attitudes towards help-seeking	64.15	10.50	0.82
Psychiatric pathogenesis beliefs	18.21	10.46	0.85
Fundamentalism	34.38	5.43	0.88
Psychological distress	4.03	3.29	0.88
Religious support	84.03	13.94	0.94

*This table shows the mean, standard deviation, range, and Cronbach Alpha for each of our questionnaires, which were used in the multiple linear regression.*

**TABLE 2:** Multiple linear regression fixed effect parameter estimates using attitudes towards seeking mental health services as criterion.

		Unstandardised coefficients		Standardised coefficients			95% confidence interval for B	
	Model	B	Std. error	Beta	*t*	*p* value	Lower	Upper
1	Intercept	3.428	0.168	0.000	20.4	<0.001	3.097	3.759
	Not Male | Male^1^	–0.1466	0.062	–0.149	–2.37	0.0188	–0.269	–0.0245
	Age	–0.0005	0.0019	–0.0157	–0.251	0.8023	–0.0043	0.0033
	Religiosity	0.0030	0.0036	0.0532	0.842	0.4008	–0.0041	0.0101
2	Intercept	3.871	0.2439	0.000	15.87	<0.001	3.39	4.352
	Not Male | Male^1^	–0.1224	0.0575	–0.125	–2.13	0.0343	–0.236	–0.0091
	Age	–0.0024	0.00182	–0.077	–1.30	0.196	–0.0060	0.0012
	Religiosity	0.0025	0.00390	0.044	0.638	0.524	–0.0052	0.0102
	Psychiatric pathogenesis beliefs	–0.1108	0.02280	–0.305	–4.859	<0.001	–0.156	–0.0659
	Fundamentalism	–0.0865	0.0417	–0.139	–2.076	0.0389	–0.169	–0.0044
	Psychological distress	–0.0250	0.00792	–0.195	–3.158	0.0018	–0.041	–0.0094
	Religious support	0.0042	0.00203	0.139	2.076	0.0390	0.0002	0.0082

*Model 1: F(3,248) = 1.99, p = 0.117, R^2^ = 0.012. Model 2: F(7,243) = 9.64, p < 0.001, R^2^ = 0.195. ΔR^2^ = 0.183.*

*^1^Given that only 5 participants self-reported as non-binary, gender was dichotomised into male and not male to aid interpretation of the model parameters. Here, not male is the reference set to 0 against which male is compared in order to calculate the coefficients.*

## Discussion

The current study aimed to investigate the relative contributions of the following variables in predicting Evangelical Christians’ attitudes towards seeking mental health help: (i) psychiatric pathogenesis beliefs that mental illness is caused by immoral or sinful living, (ii) religious support, (iii) psychological distress, and (iv) religious fundamentalism. Based on existing literature coming mainly from American Evangelicals, it was hypothesised that all but religious support would negatively predict help-seeking attitudes in our sample of United Kingdom Christians. Our results revealed support for our hypotheses in that higher fundamentalism, psychological distress, and psychiatric pathogenesis beliefs (that mental illness is caused by immoral or sinful living) were significantly correlated with less positive attitudes towards mental health help-seeking. In contrast, religious support positively predicted help-seeking attitudes since higher religious support was correlated with better attitudes towards seeking psychotherapeutic intervention.

Regarding religious support, existing research has already shown that the social aspects of religion strongly influence one’s attitudes. Evangelical Christianity emphasises the importance of interacting with fellow believers through regular fellowship at religious services, bible studies, and prayer meetings (Bebbington, 2003; Olson, 2008). These interactions increase the likelihood of engaging with individuals who have similar world outlooks, values, and meaning-making processes (Peteet, 2019). Such congeniality leads to the affirmation of the Evangelical in-group and gives its members a strong sense of coherence and identity (Ysseldyk et al., 2010). Not only has it been shown that religious support buffers the effects of mental distress (Nooney and Woodrum, 2002), but a study by Miville and Constantine (2006) reported that feeling supported by one’s religious community is also associated with more favourable attitudes towards help-seeking. Thus, in line with our own findings, religious support seems to offer a strong sense of social identity, leading to behavioural norms associated with more positive attitudes towards seeking psychological intervention.

As for fundamentalism on the other hand, the growing body of literature has shown that Evangelicals with more fundamentalist beliefs (e.g., believing in the inerrancy of the Bible) have more negative attitudes towards mental health help-seeking. For example, Keating and Fretz (1990) found that increasing religiosity was associated with a lower likelihood of seeking psychotherapeutic intervention from secular mental health professionals. A possible explanation for this is that the bible depicts parables in which God through Jesus Christ heals people of sickness through miraculous intervention (e.g., Holy Bible, New Revised Standard Version Bible, 1989/2015, Matthew 8:16). As such, those with a fundamentalist religious orientation may also be encouraged to seek God’s healing through prayer and not through secular means, which would be equated with being a bad Christian (Trice and Bjorck, 2006). As a result, religious fundamentalism may encourage persons with mental health concerns to preferentially solicit help from their religious leaders (VanderWaal et al., 2012). Although there have been notable changes to the Christian understanding of mental illness over the past 50 years, Evangelical Christianity continues to affirm the importance of deliverance ministry, commonly known as demonic exorcism (Malia, 2001). Whilst official guidelines from mainstream churches in the United Kingdom recognise contemporary biopsychosocial models of mental illness (e.g., Church of England, 2012), other Christian communities ascribe to the hyper spiritualisation of psychological illness (Mercer, 2013), sometimes vilifying the mental health professions (Vitz, 1994). In line with our own findings, such a spiritual ontology of mental illness may worsen attitudes towards psychotherapeutic intervention, and thereby encourage believers to seek pastoral or congregational help when experiencing distress (Trice and Bjorck, 2006).

Related to the correlation between more fundamentalist doctrines and worse help-seeking attitudes are the psychiatric pathogenesis beliefs that mental illness is caused by immoral living. Given the Evangelical teaching of regeneration in which believers are expected to be born again from their old life of sin, Evangelicals believe that certain behaviours increase one’s propensity towards psychological trouble (Weaver, 2011). Research has shown that Evangelical Christians are likely to see mental health as the outworking of their inner spiritual condition and therefore have poorer attitudes towards help-seeking since these interventions are perceived to ignore the spiritual constituents (Hartog and Gow, 2005). Avent et al. (2015) conducted a qualitative interview with religious leaders whose parishioners often solicit their mental health advice and found that leaders suggest that the congregation’s difficulties may be caused by neglecting their spirituality. According to Sullivan et al. (2014), there are three prevailing attitudes among religious leaders: (i) psychological disturbances are spiritual in nature with a spiritual cure, (ii) psychological disturbances are mental in nature and have a spiritual cure, and (ii) psychological disturbances are medical conditions with both spiritual and mental cures. With evidence of religious leaders not collaborating with mental health professionals when assisting their parishioners (Stansbury et al., 2012), Christians with lived experience of mental illness can find themselves being ignored or disregarded (Lloyd and Waller, 2020). Consequently, spiritual reductionism removes the person and their supposedly sinful experience from their broader life experiences (Lloyd, 2021). In line with the current findings, psychiatric pathogenesis beliefs that mental illness is caused by immoral living is then associated with less positive attitudes towards seeking secular mental health help for purportedly spiritual conditions.

Taken together, the results of this study fit squarely within and inform the theory of planned behaviour, which can be applied to formalise how one’s attitudes inform one’s intentions to use mental health services, which are the best predictors of actual service utilisation (Ajzen, 1985, 1991). In the current study, more fundamentalist beliefs in the inerrancy of scripture would dictate the normative beliefs and subjective norms of individual believers. In believing that mental illness has a spiritual cause and a spiritual solution through divine supplication or more moral living, the normative belief is that fellow believers would view psychological distress as the result of an unchristian lifestyle. As such, within the theory of planned behaviour framework, believers are less likely to have positive attitudes and intentions towards help-seeking as this could be stigmatised and raise questions over the quality of their faith. On the flip side, a greater sense of religious support is likely to reciprocally influence one’s intentions to use mental health services since it would likely favour norms that are more supportive of service utilisation and perceived control over one’s own behaviour through supportive relationships with like-minded believers.

In terms of the clinical implications of our study, although previously overlooked within the psychological sciences and clinical practice, religion and spirituality are now recognised as important clinical variables for psychologists to consider (American Psychological Association, 2017). Within counselling and psychotherapy, religious beliefs and value systems are afforded significance, equal to an individual’s wider systems and relationships (Payne et al., 1992). Sue and Sue (1999) advocate that therapists should explore their clients’ spiritual beliefs and values just as they query about their physical and social health. Furthermore, research suggests that if the therapist fails to pay due consideration towards religious and spiritual values, then effective outcomes may be impeded, with any benefits being largely restricted (Bergin, 1980). As the findings of the present study suggest that evangelical Christians with more spiritualised aetiological understandings of mental distress (as caused by sinful or immoral living) may have lower propensity to access secular mental health services, there is need for a greater strengthening between religious leaders and mental health services. This may include community based psychoeducational workshops, which encourage both clients, and religious communities, to be receptive to various levels or forms of help. This may include promoting access to help from religious leaders but also wider religious communities, and the social capital resources these may afford, in addition to, professional mental health support where appropriate. As such, the findings of the present study underline the need for clinical and psychotherapeutic practitioners to work with both clients and faith leaders in a culturally and religiously syntonic form. We suggest that psychotherapeutic practitioners should support Evangelicals to resist a binary or exclusively spiritualised approach in relation to the causes and treatments of mental distress (spiritual versus biopsychosocial factors), whilst remaining curious, tentative, and sensitive to the clients’ own frames of reference.

That being said, the results of the current study should nonetheless be caveated by a few limitations. Firstly, the gender composition of our sample consisted of three times as many males as females. Research has shown that males tend to report more negative attitudes towards seeking mental health help than females (Gonzalez et al., 2005). It has been postulated that this gender difference may be explained, at least in part, by help-seeking’s acting as a violation of gender norms in which men may feel emasculated if they cannot independently regulate their well-being (Addis and Mahalik, 2003). Within the context of religion, research has also shown that men are more likely to hold stigmatising beliefs about mental illness (Wesselmann and Graziano, 2010) and seek more religious help from pastoral leaders compared to women (Crosby and Bossley, 2012). Taken together, men may experience a compounding effect in which both society and their religion dissuade favourable attitudes towards disclosing and seeking help for mental health issues (Hammer et al., 2013). Thus, our sample of mostly males may have influenced our results in that men may have biased the negative help-seeking attitudes, which may not have been so evident in a sample with more female Evangelicals. Secondly, self-report scales were used in this study and therefore response biases, such as socially desirable responding, may have influenced our findings (Kotera et al., 2020). As such, future research in this area could consider the use of implicit measures of attitudes towards help-seeking in Evangelical populations to mitigate the impact of response biases associated with explicit self-reports (e.g., using an implicit association test; Greenwald et al., 1998). Thirdly, there might have been hidden variables that could have predicted help-seeking over and above the indices of religiosity used in the current study, such as mental health literacy (Kola-Palmer et al., 2020), sociodemographic factors (Picco et al., 2016) or previous experience of receiving psychotherapeutic care (Liang et al., 2020). These variables were not evaluated in this study and so future research should assess their contribution to the variance in help-seeking attitudes in United Kingdom Evangelical Christians. And lastly, the cross-sectional design of our regression study precludes the establishment of causality, which could have been explored using a longitudinal design to assess how changes in our predictors would affect help-seeking attitudes over time.

In conclusion, the current study aimed to investigate the factors that may predict United Kingdom Evangelicals attitudes towards seeking psychotherapeutic intervention. It was found that whilst religious support positively predicted help-seeking attitudes, psychiatric pathogenesis beliefs that mental illness is caused by immoral or sinful living, psychological distress, and religious fundamentalism were negative predictors of help-seeking attitudes. Taken together, our findings fit within a theory of planned behaviour framework, which suggests that service utilisation can be predicted from behavioural intentions, which can in turn be predicted from Evangelicals’ attitudes towards psychotherapeutic participation. As a result of our findings, we suggest that mental health practitioners be aware of religious clients’ worldviews and the impact that this can have on their propensity to seek professional help when experiencing distress.

## Data Availability Statement

The raw data supporting the conclusions of this article will be made available by the authors, without undue reservation.

## Ethics Statement

The studies involving human participants were reviewed and approved by University of Derby Ethics Committee. The patients/participants provided their written informed consent to participate in this study.

## Author Contributions

This project formed a part of CL’s earlier research projects in this area. CL led on study conception and design, ethical approval, recruitment, and data acquisition, as well as overall supervision of the research project, from literature review to study write up, and wrote the clinical implications section of the manuscript. GR conducted the literature review and statistical analyses and wrote the initial drafts of the manuscript. All authors contributed to the manuscript revision and approved the current form of the submitted version.

## Conflict of Interest

The authors declare that the research was conducted in the absence of any commercial or financial relationships that could be construed as a potential conflict of interest.

## Publisher’s Note

All claims expressed in this article are solely those of the authors and do not necessarily represent those of their affiliated organizations, or those of the publisher, the editors and the reviewers. Any product that may be evaluated in this article, or claim that may be made by its manufacturer, is not guaranteed or endorsed by the publisher.
